# T‐cell activation–induced marker assays in health and disease

**DOI:** 10.1111/imcb.12636

**Published:** 2023-03-21

**Authors:** Chad Poloni, Cole Schonhofer, Sabine Ivison, Megan K Levings, Theodore S Steiner, Laura Cook

**Affiliations:** ^1^ Division of Infectious Diseases, Department of Medicine University of British Columbia Vancouver BC Canada; ^2^ BC Children's Hospital Research Institute Vancouver BC Canada; ^3^ Department of Surgery University of British Columbia Vancouver BC Canada; ^4^ Department of Microbiology and Immunology University of Melbourne, at the Peter Doherty Institute for Infection and Immunity Melbourne Australia; ^5^ Department of Critical Care, Melbourne Medical School University of Melbourne Melbourne Australia

**Keywords:** AIM, antigen‐specific response, CD4^+^ T cells, flow cytometry

## Abstract

Activation‐induced marker (AIM) assays have proven to be an accessible and rapid means of antigen‐specific T‐cell detection. The method typically involves short‐term incubation of whole blood or peripheral blood mononuclear cells with antigens of interest, where autologous antigen‐presenting cells process and present peptides in complex with major histocompatibility complex (MHC) molecules. Recognition of peptide–MHC complexes by T‐cell receptors then induces upregulation of activation markers on the T cells that can be detected by flow cytometry. In this review, we highlight the most widely used activation markers for assays in the literature while identifying nuances and potential downfalls associated with the technique. We provide a summary of how AIM assays have been used in both discovery science and clinical studies, including studies of severe acute respiratory syndrome coronavirus 2 (SARS‐CoV‐2) immunity. This review primarily focuses on AIM assays using human blood or peripheral blood mononuclear cell samples, with some considerations noted for tissue‐derived T cells and nonhuman samples. AIM assays are a powerful tool that enables detailed analysis of antigen‐specific T‐cell frequency, phenotype and function without needing to know the precise antigenic peptides and their MHC restriction elements, enabling a wider analysis of immunity generated following infection and/or vaccination.

## Graphical abstract

An overview of validated cell surface markers, and the stimulation time required for their optimal surface expression, to detect human antigen–specific CD4^+^ and CD8^+^ T cells using flow cytometric activation‐induced marker assays.

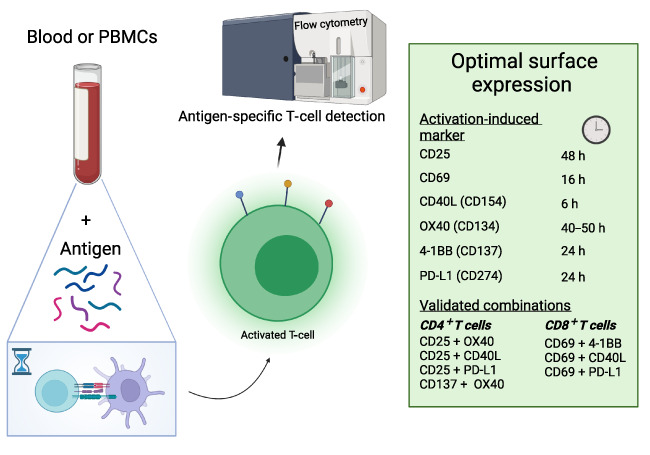

## INTRODUCTION

T cells are important mediators of the adaptive immune system, capable of carrying out coordinated, specific responses to combat infection and prevent autoinflammation. Identification of antigen‐specific T cells allows for the precise measurement of type, quality, magnitude and duration of adaptive immune responses to pathogens, tumors and vaccines. However, detection of antigen‐specific T cells is challenging owing to their low abundance in peripheral blood.

The emergence of severe acute respiratory syndrome coronavirus 2 (SARS‐CoV‐2) and the development of subsequent vaccines has emphasized the importance of measuring and monitoring adaptive immune responses. Current strategies to monitor antibody levels to define vaccine effectiveness through *in vitro* assays are helpful but fail to capture the entire spectrum of the immune response, and do not always correlate with protection against clinical disease. Here we discuss the utility of antigen‐induced marker (AIM) assays as a robust and effective method to identify and track antigen‐specific T cells *ex vivo*.

Traditionally, antigen‐specific T‐cell responses have been quantified by measuring cell proliferation, cytokine production and/or peptide–major histocompatibility complex (MHC) multimer staining. Proliferation assays are typically performed by stimulating peripheral blood mononuclear cells (PBMCs) with an antigen of interest, and quantifying cell proliferation by H^3^‐thymidine uptake or dilution of a cell‐proliferation dye, such as carboxyfluorescein succinimidyl ester (CFSE) after 4–7 days.^1^, ^2^ However, these assays require antigen stimulation that directly affects the phenotype of proliferating cells, meaning the initial frequency of antigen‐specific T cells in the original sample cannot be quantified. Proliferation assays also do not detect hypoproliferative antigen‐specific cells, such as regulatory T cells (Tregs), and are influenced by bystander activation, whereby interleukin (IL)‐2 secreted by antigen‐specific T cells drives antigen‐independent proliferation of nearby T cells.

Methods to detect antigen‐specific T cells by their cytokine production include enzyme‐linked immunospot assays (ELISPOT), cytokine capture assays and intracellular cytokine staining.^3^, ^4^, ^5^ However, these assays fail to detect the full breadth of a T‐cell response to any given antigen, as only a limited number of cytokines can be measured, with *a priori* decisions required about which cytokine‐producing cells are of interest. Further, cytokine production kinetics are complex, making it hard to capture the full magnitude of the antigen‐specific response at any given timepoint.

Staining with fluorescently conjugated peptide–MHC multimers (often termed tetramers) has been the “gold standard” to identify antigen‐specific T cells. This method has been more successful for CD8^+^ T cells than CD4^+^ T cells, as peptide‐MHC class I complexes are more stable and thus easier to produce than peptide‐MHC class II complexes. Importantly, staining with these reagents requires knowledge of the immunodominant peptides and their MHC restricting elements, which is straightforward in mice but very complex with human samples because of extensive HLA genetic polymorphisms. This limits their broad applicability for human T cells.

**Figure 1:**
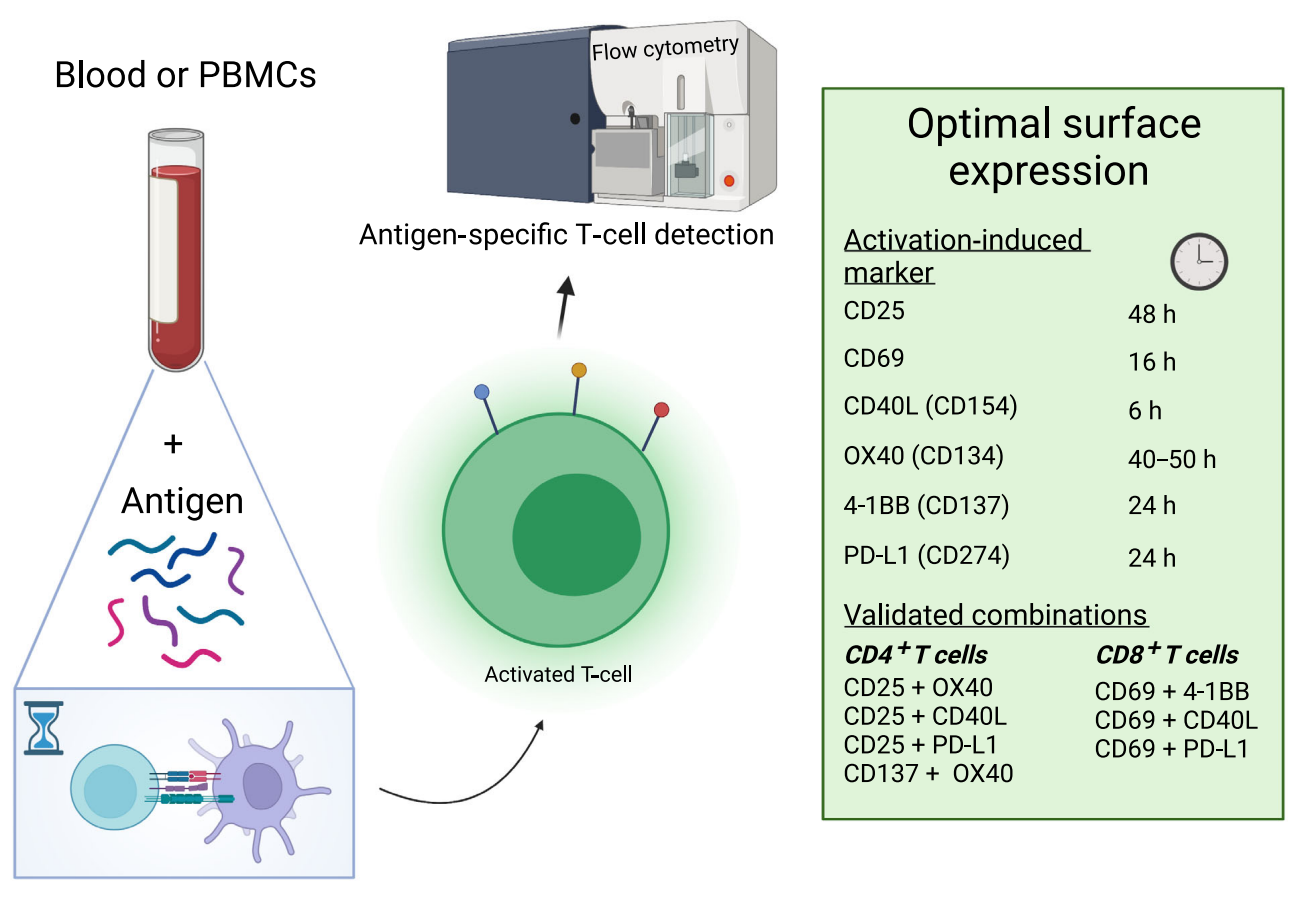
An overview of validated cell surface markers, and the stimulation time required for their optimal surface expression, to detect human antigen–specific CD4^+^ and CD8^+^ T cells using flow cytometric activation‐induced marker (AIM) assays. PBMC, peripheral blood mononuclear cell; PD‐L1, programmed death‐ligand 1.

By contrast, flow cytometric activation‐induced marker (AIM) assays allow for simultaneous detection and phenotyping of antigen‐specific T cells by staining cell‐surface markers upregulated following recognition of cognate antigen (Figure 1).^6^, ^7^ These assays are typically performed using whole blood or PBMCs, with autologous antigen‐presenting cells processing and presenting the antigens added to wells, meaning no prior knowledge of epitopes and their MHC restriction is needed. With the appropriate selection of surface markers and time‐course stimulation, AIM assays can more easily identify and phenotype a wider breadth of antigen‐specific T‐cell responses than current proliferation or cytokine‐secretion assays.

### Cell surface receptors validated for AIM assays with human blood/PBMCs

Numerous cell surface receptors have been identified and authenticated as being able to identify human antigen–specific CD4^+^ and CD8^+^ T cells. However, a variety of marker combinations and stimulation conditions have been published, making reproducibility and standardization difficult. Here we provide a summary and discussion of the advantages and disadvantages of these approaches.

CD25 (IL‐2Rα) is one of the most commonly used cell surface receptors to identify antigen‐specific CD4^+^ T cells. It is important to note that CD25 must always be used in tandem with another marker to define antigen‐specific cells, as it is constitutively present on 0.5–6% of CD4^+^ T cells, being the Tregs.^8^, ^9^ Initial upregulation of CD25 is a result of TCR signaling *via* the nuclear factor‐kappa B pathway, with frequency directly correlated with signaling strength.^9^ Maintenance of CD25 is mediated by IL‐2–induced STAT5 (signal transducer and activator of transcription 5) signaling, which peaks at 48 h following activation.^9^ CD25 is also upregulated on CD8^+^ T cells following activation; however, it has not been validated to define antigen‐specific CD8^+^ T cells and, of note, increased levels of CD8^+^CD25^+^ T cells have been reported in some elderly individuals (mean age 75 years).^10^ Instead, the type II C‐lectin receptor, CD69, is more commonly used to identify antigen‐specific CD8^+^ T cells and was the original marker in this field; indeed, an alternate name for CD69 is activation inducer molecule.^11^, ^12^ CD69 is upregulated *via* TCR signaling, and its surface expression peaks at 16 h after activation on CD8^+^ T cells.^13^ However, signaling through α_4_β_1_ integrin or *via* IL‐2, IL‐4, IL‐12, interferon‐(IFN)α and IFN‐γ cytokines through their receptors will increase surface expression of CD69, meaning it also needs to be used in tandem with at least one other marker.^13^, ^14^ Of note, Tregs are more sensitive to TCR‐independent, CD69 induction signals than non‐Tregs, which may lead to false identification of antigen‐specific Tregs.^15^

CD40 ligand (CD40L; CD154), a member of the tumor necrosis factor (TNF) superfamily, was one of the first surface receptors to be used and validated for AIM assays. CD40L was originally described for CD4^+^ T cells and later expanded for use with CD8^+^ T cells.^6^, ^7^, ^16^ CD40L is expressed transiently by CD4^+^ and CD8^+^ T cells in response to antigen stimulation, and CD40L^+^ T cells produce large amounts of cytokines, IL‐2, IL‐4, IL‐5, IL‐10, IFN‐γ and TNF depending on the stimulus.^6^, ^7^ However, following binding to its cognate receptor, CD40, CD40L is rapidly downregulated within 6 h of activation.^17^ Activation probably causes CD40L to cycle between the cell surface and cytoplasm as it has been detected intracellularly within HIV‐antigen‐stimulated IFN‐γ‐producing cells in HIV‐infected patients.^18^ To combat this downregulation, a fluorescent anti‐CD40L antibody can be added for the duration of *in vitro* stimulation. This method was first described by Chattopadhyay *et al*. where, although CD40L surface expression peaked at 6 h, the protein–antibody complex could be detected up to 24 h post stimulation.^6^ This technique has been demonstrated on both fresh and previously frozen PBMCs. Of note, monensin, an inhibitor of protein export from the Golgi complex, is required to stabilize the internalized CD40L protein–antibody complexes while use of brefeldin abrogates detection.^6^ Alternatively, anti‐CD40 blocking antibodies can be used to prevent internalization of CD40L, resulting in similar expression kinetics as the Chattopadhyay method.^7^ In addition, costaining for additional activation markers, such as CD69, can increase assay sensitivity.^19^

More recently, OX40 (CD134), another member of the TNF receptor superfamily, has been validated in combination with CD25 to identify antigen‐specific CD4^+^ T cells in AIM assays.^20^ Unlike other activation‐induced markers, OX40 is not expressed on nonactivated T cells in humans, but similarly its surface expression is driven by TCR signaling, with expression levels peaking at about 48 h after activation.^21^ OX40 expression levels are tightly controlled by TCR signaling, with additional fine‐tuning *via* CD28, IL‐2 and IL‐4 signaling.^22^ OX40 signaling plays a vital role in T‐cell expansion and survival, as well as T‐helper cell differentiation. Unlike CD40L, which is biased toward expression on T‐helper type 1 (Th1) cells, OX40 is expressed equally on all Th cells, including Tregs, potentially reducing bias when analyzing antigen‐specific T cells.^16^, ^23^, ^24^ It is important to note that OX40 is highly expressed on murine FOXP3^+^ Tregs, making it unsuitable for analysis of peripheral murine CD4^+^ T cells.^25^

The CD25/OX40 AIM assay has been validated clinically using fresh, heparinized whole blood within 24 h of collection, with analysis following 40–50 h of antigen stimulation.^20^, ^26^ It is important to note that this whole blood assay is validated for blood collected in tubes containing heparin (sodium or lithium), and although a comprehensive testing of all anticoagulants has not been performed, it is known that ethylenediaminetetraacetic acid and acid–citrate–dextrose inhibit marker upregulation due to inhibiting Ca^2+^ influx; however, PBMCs can be used after isolation from any of these blood collection tubes.^27^ This assay correlates with cell proliferation, intracellular cytokine and tetramer staining measures of antigen‐specific T cells, as well as serology.^26^, ^28^ In comparison to CD69/CD40L and IFN‐γ intracellular cytokine staining assays, the CD25/OX40 AIM assay detects a larger population of antigen‐specific CD4^+^ T cells, including rare, noncytokine‐producing T‐cell subsets such as T follicular helper (Tfh) cells.^26^, ^29^ This suggests that CD25/OX40 is currently the best published method to capture the full breath of antigen‐specific CD4^+^ T‐cell responses.

Another validated marker for AIM assays is the TNF receptor superfamily member, 4‐1BB (CD137). Activation of T cells *via* TCR drives upregulation of 4‐1BB, which through binding its ligand 4‐1BBL (CD137L) on antigen‐presenting cells drives T‐cell proliferation and cytokine production.^30^, ^31^ Notably, 4‐1BB can be paired with CD69 to detect antigen‐specific CD8^+^ T cells^32^, with optimal surface expression at 24 h after activation.^33^ Although best validated for detecting CD8^+^ T cells, 4‐1BB is also expressed on CD4^+^ T cells as early as 6 h following activation, and combined with a lack of CD40L expression, could detect stable human Tregs in long‐term culture.^34^, ^35^ Therefore, within 6 h of activation, when CD40L is only upregulated on nonregulatory cells, the combination of 4‐1BB and CD40L might be able to differentiate Treg and T‐effector antigen‐specific CD4^+^ T‐cell populations.

Programmed death‐ligand 1 (PD‐L1 or CD274) is another early activation marker that can be used in AIM assays. PD‐L1 functions to suppress autoimmunity and, importantly, plays a pivotal role in suppressing antitumor immunity by signaling to programmed cell death protein 1 (PD‐1) on antitumor T cells. As such, PD‐L1/PD‐1 signaling is an immunotherapy drug target in various cancers.^36^ When combined with CD25, PD‐L1 was found to be equivalent to OX40 in detecting antigen‐specific Tfh cells.^29^ In addition, while CD25/OX40 staining captures the full range of T‐cell subtypes, OX40/PD‐L1 staining has been described to exclude Tregs, thus providing a way to discriminate between Treg and non‐Tregs.^37^ It is important to note that PD‐L1 signaling prevents Th1 polarization, directs Th17 differentiation and induces an IFN‐γ‐anergic phenotype.^38^ As such, PD‐L1 may identify an anergic and nonfunctional antigen‐specific T‐cell subset, especially when characterizing antitumor responses.

### AIM assays using tissue and nonhuman samples

AIM assays have also been adapted for use with nonhuman samples, primarily mouse and nonhuman primates, and with tissue samples. Notably, CD40L has been validated as a marker of murine antigen‐specific circulating CD4^+^ T cells, with CD25 and OX40 also validated for splenocytes and Tfh cells, with detection after 18 h of stimulation.^20^, ^39^ Similar to human T cells, murine T cells rapidly downregulate CD40L, necessitating the use of CD40 blocking antibody or the addition of anti‐CD40L antibody to culture.^40^ The combination of CD25 and OX40 to detect antigen‐specific CD4^+^ T cells seems to have the widest validation, including macaque blood and for lymph node and tonsil samples from humans and macaques.^20^, ^29^, ^39^, ^41^, ^42^, ^43^, ^44^ Although AIM assays for murine CD8^+^ T cells are not as well characterized, it is known that CD69 and 4‐1BB surface expression are induced upon activation of murine T cells. Following stimulation, CD69 expression peaks between 24 and 48 h, although expression levels decrease with 2‐month‐old mice compared with 24‐month‐old mice,^45^ and up to 5% of non‐antigen‐specific T cells express CD69 after stimulation.^40^ 4‐1BB can be detected on murine CD8^+^ T cells 12 h after stimulation, with expression peaking at about 24 h.^46^ Of note, AIM assays using human bone marrow, spleen, lung, lung‐associated and gut‐associated lymph node samples have defined antigen‐specific CD4^+^ T cells as CD40L^+^OX40^+^ or 4–1BB^+^OX40^+^ or 4–1BB^+^CD40L^+^ and antigen‐specific CD8^+^ T cells as 4–1BB^+^CD25^+^.^47^ In addition, while circulating human T cells in blood are CD69^neg^, tissue‐resident memory T cells coexpress CD69 and CD103, therefore CD69 should be avoided when using tissue samples (reviewed by Thome and Farber^48^). Because of species‐ and tissue‐specific differences in activation‐induced marker utility and kinetics, assays with nonhuman and tissue samples require careful validation.

### Characterization of the antigen‐specific T‐cell response

With the rapid advancement of high‐dimensional flow cytometry, it is possible to pair identification of antigen‐specific T cells with phenotypic analysis and proliferation assays.^49^ This approach utilizes previously identified surrogate cell surface markers of interest to characterize or sort the antigen‐specific T‐cell response, allowing for deeper insights into adaptive immune responses.

Detection of chemokine receptors on the cell surface has been widely used to identify subsets of T cells *ex vivo*. When coupled with AIM assays, phenotyping *via* chemokine receptors becomes a powerful tool in identifying the cell subsets that constitute an antigen‐specific response. Notably, naïve, effector memory, effector memory re‐expressing CD45RA (TEMRA) and central memory T cells can be easily identified *via* their expression of CD45RA, CD45RO and CCR7 (naïve: CCR7^+^CD45RA^+^CD45RO^neg^; effector memory: CCR7^neg^CD45RA^neg^CD45RO^+^; TEMRA:CCR7^neg^CD45RA^+^CD45RO^neg^; central memory: CCR7^+^CD45RA^neg^CD45RO^+^).^50^

CD4^+^ T cells can be further broken down into T helper (Th) subsets by their surface expression of chemokine receptors. Notably, CXCR3 expression can be used to identify Th1 cells, with the addition of CCR4 and CCR6 used to identify IFN‐γ‐ and IL‐17A‐producing Th17.1 cells (Th1 cells are CCR4^neg^CCR6^neg^).^51^, ^52^, ^53^ In addition, the alternate combinations of these markers can be used to identify Th2 (CXCR3^neg^CCR4^+^ CCR6^neg^) and Th17 cells (CXCR3^neg^CCR4^+^ CCR6^+^), with Th9 cells proposed to be CXCR3^+/−^ (losing CXCR3 during acute reaction) CCR4^neg^CCR6^+^ and Th22 cells to be CCR4^+^CCR6^+^CCL10^+^.^54^, ^55^, ^56^, ^57^, ^58^ When coupled with the CD25/OX40 assay, expression of the ecto‐nucleoside triphosphate diphosphohydrolase CD39 identifies a cell population highly enriched (> 80%) for Tregs,^59^ noting that a single‐nucleotide polymorphism (rs10748643) determines surface expression levels of CD39.^60^ However, CD39 alone also identifies a second population that is FOXP3^neg^ and produces high levels of IFN‐γ and IL‐17A.^61^ CD39^+^ Treg identification may prove to be a useful alternative to intracellularly staining for FOXP3, especially if CD25 is included in the assay.^24^ In addition, it is possible to identify circulating Tfh (cTfh) based on CXCR5 and PD‐1 expression.^29^ Of note, CXCR5‐expressing CD8^+^ T cells important for the control of autoantibody generation in the germinal center and for shaping the antibody response to viral infections.^62^, ^63^, ^64^

It is important to note that while studies have shown chemokine receptors to be a useful alternative to cytokine detection, proper validation should be carried out to verify findings. This is especially important in disease cohorts, where alterations to chemokine receptor expression may occur *in vivo*. Care must also be taken to validate *in vitro* stimulation–induced changes in lineage‐defining surface markers during the time course of the assay, with one method for this being stimulation of sorted *ex vivo* cell populations.^49^ Prolonged stimulation should also be carefully optimized as this may cause changes in surface receptor expression, particularly through inducing cell proliferation and/or death. Validation can be achieved through various methods, including intracellular staining or cell sorting and qualitative multiplex single‐cell RT‐PCR of transcription factors.^65^ AIM assays are also particularly useful for repertoire analysis of TCR sequences as viable antigen‐specific T cells can be isolated prior to cell division occurring.^66^

### Clinical applications of AIM assays

#### Infectious diseases

Use of activation‐induced markers to identify antigen‐specific T‐cell populations has many potential uses, including direct clinical applications. In the context of vaccines, AIM assays can be used to determine vaccine effectiveness, track T‐cell responses and potentially inform on when and whether additional doses are needed. Further, AIM assays have the potential to detect pathogenic responses in a clinical setting, provide clinicians with additional information, allow for more informed decisions and ultimately improve patient outcomes.

One area where AIM assays may be of use clinically is in the detection of T‐cell responses to *Mycobacterium tuberculosis* (MTB) infection. MTB infection is often chronic and can be latent for years before becoming active and causing clinical illness. Screening for latent MTB is recommended for people at risk of MTB acquisition and for those with immune compromise who are at a high risk of disseminated and/or lethal MTB reactivation. Traditional MTB detection relies either on tuberculin skin testing, which measures induration at the site of intradermal injection of an MTB‐purified protein derivative, or on IFN‐γ release assays. However, MTB infection can be comorbid with immunocompromise, particularly advanced HIV‐infection, which reduces the sensitivity of these methods. AIM assays can detect a wider range of MTB‐specific T cells than IFN‐γ release assays, including those that do not produce IFN‐γ. The CD25/OX40 AIM assay was shown to perform similar to tuberculin skin testing and QuantiFERON‐TB Gold In‐Tube (IFN‐γ release assay) and Xpert MTB/RIF tests in detecting MTB‐specific CD4^+^ T cells in both HIV‐infected and uninfected participants.^67^, ^68^ Both CD25/OX40 and CD69/CD40L AIM assays can detect latent MTB infection in HIV‐infected individuals.^69^ Moreover, using the CD25/OX40 assay in combination with IFN‐γ release assays improves prediction of progression from latent to active MTB infection and can detect latent MTB infection in patients receiving TNF inhibitors, a group with high false negatives using conventional tests.^70^, ^71^

AIM assays have also enabled discovery research into infectious diseases, furthering our understanding of acute and memory T‐cell kinetics following an infection. For example, AIM assays using OX40/4‐1BB markers have been used to explore the cross reactivity of dengue‐specific effector memory CD4^+^ T cells to Zika virus antigens.^72^ The CD25/OX40 AIM assay, among other techniques, has also been used to explore why some patients suffer from recurrent group A *Streptococcus* infections, leading to the characterization of recurrent group A *Streptococcus* tonsillitis as an immune‐susceptibility disease with insufficient group A *Streptococcus* germinal center Tfh cells.^73^ Furthermore, AIM assays have been used to explore and characterize HIV‐Gag‐specific CD4^+^ and CD8^+^ T‐cell populations in HIV‐infected patients.^18^, ^74^, ^75^

A more recent application of AIM assays is in the examination of T‐cell responses to novel treatments. For example, the CD25/OX40 AIM assay was used to investigate the effects of fecal microbiota transplant for recurrent *Clostridioides difficile* infection on T‐cell immunity to *C. difficile* toxins. Patients with severe recurrent *C. difficile* infection had decreased toxin‐specific Th17 cells compared to healthy controls, which was increased following treatment.^76^, ^77^ In addition, AIM assays using various combinations of CD69, CD25, OX40, PD‐L1 and CD40L have been used to identify defects in Tfh responses to hepatitis B virus antigens in patients with chronic hepatitis B virus infection.^78^ Similarly, AIM assays using PD‐L1, 4‐1BB and CD69 have demonstrated that broadly neutralizing antibody therapy in HIV‐infected patients during antiretroviral therapy interruption is associated with enhanced HIV‐specific CD4^+^ T‐cell responses.^79^

#### Autoimmune diseases

Autoimmune diseases are complex, multifactorial disorders that often have numerous triggers. AIM assays have been used to understand and elucidate the contribution of T cells to the pathogenesis of these diseases. Many autoimmune diseases are associated with dysregulated T‐cell targeting of host proteins and are treated with immunosuppressive therapies. AIM assays allow for analysis of T‐cell function in patients with autoimmune diseases and responses to therapies.

Current treatment of autoimmune diseases frequently involves anti‐cytokine therapies, which carry risks of opportunistic infections. The CD25/OX40 AIM assay was used to explore the longitudinal effects of anti‐TNF drugs on T‐cell function over an 8 year period in patients with chronic rheumatic diseases.^80^ In addition, several autoimmune diseases, such as rheumatoid arthritis and multiple sclerosis, are often treated with anti‐CD20 drugs (e.g. Rituximab), which greatly decrease B‐cell activity and humoral immunity. As such, protection from infection in these patients greatly depends on cellular immunity provided by T cells. AIM assays can be used to monitor and explore responses to vaccines in these patients, to ensure that cellular immunity is improved following vaccination.^81^ More recently, clinical trials of type 1 diabetes have used AIM assays to both monitor disease and assess the effects of new therapies. Specifically, the CD25/OX40 AIM assay was used in a phase I/II trial using pluripotent stem cell–derived pancreatic endoderm cells^82^ and a pilot study treating adult patients with new‐onset type 1 diabetes with the anti‐IL‐12/23 monoclonal antibody ustekinumab.^83^ This assay is now being used to monitor diabetogenic antigen‐specific T cells in adults (Canadian phase II/III study: NCT03941132) and adolescents (UK phase II study: ISRCTN14274380) being treated with ustekinumab.^84^ These data demonstrate AIM assays as a valuable tool in human clinical studies.

Inflammatory bowel disease is a chronic autoinflammatory condition, where CD4^+^ T cells with specificity to commensal bacterial antigens are known to contribute to pathogenesis. Using CD40L as a marker for microbiota‐specific CD4^+^ T cells, Hegazy *et al*.^85^ determined that reactivity to intestinal bacteria is present in both healthy controls and patients with inflammatory bowel disease, with the majority of these being Th17 cells, which had increased IL‐17A production compared with healthy controls. Similarly, using the CD25/OX40 AIM assay, Cook *et al*.^86^ demonstrated increased bacterial flagellin–specific T cells in patients with inflammatory bowel disease compared with healthy controls, but identified that the Th17‐cell proportions were reduced in patients. Therefore, a combination of AIM assay surface markers and cytokine production may be needed to fully appreciate the role for bacterial antigen–specific Th17 cells in patients with inflammatory bowel disease.

In a similar vein, AIM assays have been used in the allergy field for the detection of allergens, treatment evaluation and treatment progression. CD40L and 4‐1BB have been used to measure allergic responses to cockroach antigens, finding associations between antigen‐specific T‐cell magnitude and phenotype with immunoglobulin E responses.^87^ The CD25/OX40 assay has also been used to identify boiled peanuts as an oral immunotherapy candidate for patients with peanut allergies^88^ and to show that imantinib‐specific T cells decreased with repeated desensitization in patients with nonhypersensitive reactions to imantinib.^89^

#### Vaccine responses

Vaccine monitoring is another major clinical use of AIM assays. Vaccine efficacy is traditionally measured by B‐cell–mediated antibody responses, as there are well‐validated antibody correlates of protection for many diseases. By contrast, we do not yet have validated correlates of protection for CD4^+^ or CD8^+^ T‐cell responses to vaccines. AIM assays allow direct characterization of antigen‐specific T‐cell responses before and after immunization,^20^, ^90^ which is particularly useful in patients with impaired humoral immunity, such as in patients receiving anti‐CD20 monoclonal antibodies or with common variable immunodeficiency, where although influenza immunization fails to produce a humoral response in patients with common variable immunodeficiency, it does produce a T‐cell response.^91^

AIM assays have been useful in vaccine development to measure effectiveness in generation of T‐cell responses. For example, CD25/OX40 and OX40/PD‐L1 assays have been used to demonstrate vaccine immunogenicity in Ebola candidate vaccines.^92^ The CD25/OX40 assay has also been used to measure T‐cell responses to a candidate enterotoxigenic *E. coli* vaccine^93^ and, with the addition of CD39, to measure contributions of regulatory and effector HIV‐specific T cells in vaccine trials.^94^, ^95^ In addition, CD25, CD69, 4‐1BB and OX40 markers have been used to compare effectiveness of malaria vaccines,^96^ with one failed vaccine candidate attributed to low humoral responses found to poorly stimulate Tfh cells.^97^ Ultimately, AIM assays can aid in vaccine development and provide context as to why certain platforms succeed, while others fail. This type of analysis is critical when developing new vaccines to emerging pathogens and is being heavily employed with evaluation of the novel SARS‐CoV‐2 vaccines.

### Detecting SARS‐CoV‐2–specific T cells following infection and vaccination

In response to the COVID‐19 pandemic, there was a rapid development of vaccines, which created an immediate need to evaluate and track adaptive immune responses. especially with the continued emergence of new SARS‐CoV‐2 variants. AIM assays quickly dominated the COVID‐19 T‐cell literature, providing immediate insights into SARS‐CoV‐2–specific responses. These findings have been summarized in Table 1.

**Table 1:** Summary of studies using AIM assays to interrogate SARS‐CoV‐2–specific T‐cell responses.

Main findings	Date published	AIM markers	Cell type	Stimulus	Duration of assay (h)	Reference
T cells recognize SARS‐CoV‐2 M, N and S peptides in 40–60% of unexposed individuals	May 2020	CD4^+^: OX40 + 4‐1BB CD8^+^: CD69 + 4‐1BB	PBMCs (1 × 10^6^ cells/well, 96‐well plate)	Spike, matrix and nucleocapsid peptide megapool (1 μg mL^−1^)	24	^99^
Common cold coronavirus–specific CD4^+^ T cells are cross‐reactive to COVID‐19	October 2020	CD4^+^: OX40 + 4‐1BB	PBMCs (1 × 10^6^ cells/well, 96‐well plate)	Spike peptide megapool (1 μg mL^−1^)	24	^109^
Coordinated T‐cell and antibody responses associate with milder COVID‐19, with impairments related to aging	November 2020	CD4^+^: OX40 + 4‐1BB + CD40L CD8^+^: CD69 + 4‐1BB	PBMCs (1 × 10^6^ cells/well, 96‐well plate)	Spike peptide megapool (1 μg mL^−1^)	24	^32^
Memory T cells persist for 3 months after mild COVID‐19 infection	January 2021	CD4^+^: ICOS + CD40L	PBMCs (4 × 10^6^ cells mL^−1^)	Full‐length spike protein (2 μg mL^−1^)	20	^110^
SARS‐CoV‐2–specific Th1 and Tfh cell responses following first vaccination dose correlate with antibody levels after second dose	September 2021	CD4^+^: CD200 + CD40L CD8^+^: 4‐1BB (+ IFN‐γ)	Frozen PBMCs (1 × 10^6^ cells/well, 96‐well plate), rested overnight	Spike peptide megapool (1 μg mL^−1^) + anti‐CD28/CD49b	24	^101^
BNT126b2 vaccination induces SARS‐CoV‐2 omicron variant–specific CD4^+^ and CD8^+^ T cells	January 2022	CD4^+^: CD69 + CD40L CD8^+^: CD69 + CD40L	Frozen PBMCs (1 × 10^6^/well, 96‐well plate), rested for 4 h	15mer OR 20mer overlapping spike peptide pools (1 μg mL^−1^)	12	^111^
Patients with COVID‐19 have defective spike‐specific Tfh responses following mRNA vaccination	February 2022	CD4^+^: CD25, OX40	Fresh PBMCs (1 × 10^6^ cells mL^−1^), 24‐well plate	Spike overlapping peptide pool (1 μg mL^−1^)	48	^112^
Vaccine‐induced SARS‐CoV‐2 spike‐specific T‐cell responses to variant‐derived peptides were not significantly different from the original Wuhan strain	March 2022	CD4^+^: OX40 + 4‐1BB CD8^+^: CD69 + 4‐1BB	Frozen PBMCs (1 × 10^6^/well, 96‐well plate), rested for 4 h	Spike 15mer overlapping peptide pool or megapool (1 μg mL^−1^)	20	^102^
Previously infected, vaccinated individuals have a distinct subset of CD127^low^ IL‐10^+^ type 1 regulatory T cells	April 2022	CD4^+^: CD69 + 4‐1BB or CD69 + CD40L	Frozen PBMCs (5 × 10^6^ cells mL^−1^), polystyrene tubes	SARS‐CoV‐2 S, M and N peptide megapools (5 μg mL^−1^)	18	^113^
Development of the B and T cell tandem lymphocyte evaluation (BATTLE) assay for concurrent detection of spike‐specific B‐ and T cells	June 2022	CD4^+^: CD69 + OX40 CD8^+^: CD25 + CD69	Frozen PBMCs (5 × 10^6^ cells mL^−1^), polystyrene plates	Overlapping spike peptides (1 μg mL^−1^)	24	^114^
mRNA, Novavax and adenovirus‐based SARS‐CoV‐2 vaccines induce spike‐specific CD4^+^ and CD8^+^ T cells in most individuals 6 months after the first dose. mRNA and Novavax vaccines induce a greater magnitude of spike‐specific CD4^+^ T cells than natural infection	July 2022	CD4^+^: OX40 + CD40L + CD137 CD8: CD69 + CD137	Frozen PBMCs in 96‐well plates	SARS‐CoV‐2 peptide megapool (1 μg mL^−1^)	24	^115^
SARS‐CoV‐2–specific CD4^+^ T‐cell responses were reduced in preschool‐aged children and increased with age	July 2022	CD4^+^: CD69 + OX40	Frozen PBMCs in 96‐well plates	SARS‐CoV‐2 and spike peptide megapools (1 μg mL^−1^)	24	^116^
T‐cell immunity 7 months after low‐dose mRNA vaccination is similar to infection‐driven immunity	September 2022	CD4^+^: OX40 + 4‐1BB CD8^+^: CD69 + 4‐1BB	PBMCs (1 × 10^6^ cells/well, 96‐well plates)	SARS‐CoV‐2 peptide megapool (1 μg mL^−1^)	24	^109^

COVID‐19, coronavirus disease 2019; IFN, interferon; IL, interleukin; mRNA, messenger RNA; PBMC, peripheral blood mononuclear cell; SARS‐CoV‐2, severe acute respiratory syndrome coronavirus 2; Tfh, T follicular helper; Th1, T helper type 1.

The Sette and Crotty groups were among the first to publish data from AIM assay characterization of COVID‐19 T‐cell immunity, discovering that natural infection–induced CD4^+^ and CD8^+^ T cells have a half‐life of 3–5 months and that the magnitude of CD4^+^ and CD8^+^ T‐cell responses negatively correlates with disease severity.^32^, ^98^ These groups also identified SARS‐CoV‐2–specific T cells (from stimulation with HLA‐I– and HLA‐II–predicted peptide megapools) in 40–60% of uninfected individuals, hypothesizing that this was due to cross‐reactivity between SARS‐CoV‐2 and previously circulating coronaviruses.^99^ They subsequently used AIM assays to map the immunodominant T‐cell–activating epitopes, generating megapools that were provided to the research community and used as a stimulus in AIM assays.^100^ Peptide pools spanning the entirety of the matrix (M), nucleocapsid (N) and spike (S) proteins are also commercially available. The readily available peptide products have allowed for rapid and reproducible evaluation of COVID‐19 vaccine–induced T‐cell responses to both the ancestral and variant viral proteins.

When coupled with phenotypic analysis, AIM assays can reveal T‐cell subsets that have predictive capability. This is especially important when making decisions about redosing populations, especially those who are immunocompromised, as these T‐cell subsets can act as early warning signs of waning antibody levels. AIM assays using CD40L have identified that, following COVID‐19 vaccination, Th1 and Tfh cells are expanded, with the latter correlating with protective humoral immunity.^101^ A study of long‐term immunity to SARS‐CoV‐2 found that increased levels of S receptor–binding domain–specific CD25^+^OX40^+^ CD4^+^ T cells associated with higher neutralizing antibody titers, with single‐cell transcriptomics revealing a heterogenous population.^24^

More recently, AIM assays have been used to determine vaccine‐induced protection against omicron and other emerging SARS‐CoV‐2 variants. The CD25/OX40 assay indicated that individuals receiving ChAdOx‐1S, Ad26.COV2.S, mRNA‐1273 or BNT162b2 vaccines had no significant differences in the level of antigen‐specific T cells when stimulated with ancestral spike protein as compared with delta, beta and omicron variant spike proteins, while antibody levels against omicron were significantly decreased.^102^ Further, 77% of vaccinated individuals were found to have omicron cross‐reactive CD4^+^ T cells, using CD69 and CD40L AIM assays.^103^ Most notably, when analyzed independently, T‐cell immunity induced by all vaccine types was highly conserved against all variants, with greater than 80% of CD4^+^ and CD8^+^ memory responses conserved against omicron.^104^

### Challenges and future directions

Although most studies show robust vaccine‐induced T‐cell immunity, the lack of standardization and variety of AIM assay used makes data interpretation challenging. A diverse combination of cell surface markers in AIM assays have been used to track COVID‐19 vaccine–induced T‐cell responses. Most commonly, CD25, CD40L and OX40 are used for CD4^+^ T cells and CD69 and 4‐1BB are used for CD8^+^ T cells. However, the differing combinations seem to yield varying results when evaluating the phenotypes comprising the response. For example, studies using CD69, CD40L and CD200 as activation markers for CD4^+^ T cells identify Th1 cells comprising the majority of the T‐cell response.^102^, ^105^ Interestingly, the use of CD40L may be skewing these results. In fact, CD40L^+^ cells have been shown to be enriched for IFN‐γ‐producing Th1 cells.^18^, ^106^ Alternatively, Th17‐like cells, identified using the activation marker CD69 alone, were found to be predominant in the nasal mucosa following SARS‐CoV‐2 mRNA vaccination.^107^ This highlights the heterogeneous nature of antigen‐specific T cells when using different combinations of activation markers. These data indicate that the best practice is to include multiple activation markers when evaluating vaccine‐induced T‐cell responses.

## CONCLUSIONS

AIM assays have emerged as a quick and robust method to evaluate antigen‐specific T‐cell responses. When coupled with phenotyping, antigen‐specific CD4^+^ and CD8^+^ T cells can be resolved to the single‐cell level. Although most AIM assays are comparable in detecting the overall magnitude of T‐cell responses, it is clear they differ in the detection of specific subsets. Careful attention must be paid when choosing the combination of surface markers to assays, as this will determine the breadth of the antigen‐specific T‐cell response being detected. Standardization is clearly needed in the field, with such work already underway as an initiative by the Canadian COVID Immunity Task Force (CITF) and Canadian Autoimmunity Standardization Core (CAN‐ASC). Furthermore, AIM assays may provide public health benefits, especially when evaluating new vaccines and tracking future outbreaks.^108^ Such standardization will be essential for regulatory bodies to consider the addition of T‐cell AIM assays when evaluating new vaccines. When used correctly, AIM assays can provide deep insights into responses to pathogens, vaccine‐induced immunity, allergies and autoimmunity.

## CONFLICT OF INTEREST

MKL received research funding from Bristol‐Myers Squibb and Takeda for work unrelated to this review. TSS received research funding from Merck, Sanofi Pasteur, Rebiotix, Seres, NuBiyota, Edesa, Summit and Pfizer for work unrelated to this review. The other authors have no commercial or financial interests to report.

## AUTHOR CONTRIBUTIONS

**Chad Poloni:** Conceptualization; writing – original draft. **Cole Schonhofer:** Writing – original draft. **Sabine Ivison:** Conceptualization; writing – review and editing. **Megan K Levings:** Conceptualization; supervision; writing – review and editing. **Theodore S Steiner:** Conceptualization; supervision; writing – review and editing. **Laura Cook:** Conceptualization; supervision; writing – review and editing.

## Data Availability

Data sharing not applicable to this article as no datasets were generated or analysed during the current study.
